# A Randomized Multicentre Phase II Trial Comparing Adjuvant Therapy in Patients with Interferon Alpha-2b and 5-FU Alone or in Combination with Either External Radiation Treatment and Cisplatin (CapRI) or Radiation alone regarding Event-Free Survival – CapRI-2

**DOI:** 10.1186/1471-2407-9-160

**Published:** 2009-05-26

**Authors:** Angela Märten, Jan Schmidt, Jennifer Ose, Sabine Harig, Ulrich Abel, Marc W Münter, Dirk Jäger, Helmut Friess, Julia Mayerle, Guido Adler, Thomas Seufferlein, Thomas Gress, Roland Schmid, Markus W Büchler

**Affiliations:** 1 Department of Surgery, University of Heidelberg, Im Neuenheimer Feld 110, 69120 Heidelberg, Germany; 2 National Center for Tumor Diseases, Im Neuenheimer Feld 350, 69120 Heidelberg, Germany; 3 Department of Radiation Oncology, University of Heidelberg, Im Neuenheimer Feld 410, 69120 Heidelberg, Germany; 4 Department of Surgery, Technische Universität München, Ismaningerstrasse 22, 81675 Munich, Germany; 5 Department of Medicine A, Ernst-Moritz-Arndt-Universität Greifswald, Friedrich-Loeffler-Str. 23a, 17475 Greifswald, Germany; 6 Department of Gastroenterology, University of Ulm, Robert Koch Strasse 8, 89081 Ulm, Germany; 7 Department of Gastroenterology, University of Halle, Ernst-Grube-Str. 40, 06120 Halle (Saale), Germany; 8 Department of Gastroenterology, University of Marburg, Baldingerstrasse, 35043 Marburg, Germany; 9 Department of Gastroenterology, Technische Universität München, Ismaningerstrasse 22, 81675 Munich, Germany; 10 Department of Medical Biometry, University of Heidelberg, Im Neuenheimer Feld 405, 69120 Heidelberg, Germany

## Abstract

**Background:**

The 5-year survival of patients with resected pancreatic adenocarcinoma is still unsatisfying. The ESPAC-1 and the CONKO 001 trial proofed that adjuvant chemotherapy improves 5-year survival significantly from approximately 14% to 21%. In parallel, investigators from the Virginia Mason Clinic reported a 5-year survival rate of 55% in a phase II trial evaluating a combination of adjuvant chemotherapy, immunotherapy and external beam radiation (CapRI-scheme). Two other groups confirmed in phase II trials these results to a certain extent. However, these groups reported severe gastrointestinal toxicity (up to 93% grade 3 or 4 toxicity). In a randomized controlled phase III trial, called CapRI, 110 patients were enrolled from 2004 to 2007 in Germany and Italy to check for reproducibility. Interestingly, much less gastrointestinal toxicity was observed. However, dose-reduction due to haematological side effects had to be performed in nearly all patients. First clinical results are expected for the end of 2009.

**Methods/Design:**

CapRI-2 is an open, controlled, prospective, randomized, multicentre phase II trial with three parallel arms. A de-escalation of the CapRI-scheme will be tested in two different modifications. Patients in study arm A will be treated as outpatients with the complete CapRI-scheme consisting of cisplatin, Interferon alpha-2b and external beam radiation and three cycles of 5-fluorouracil continuous infusion. In study arm B the first de-escalation will be realised by omitting cisplatin. Next, patients in study arm C will additionally not receive external beam radiation. A total of 135 patients with pathologically confirmed R0 or R1 resected pancreatic adenocarcinoma are planned to be enrolled. Primary endpoint is the comparison of the treatment groups with respect to six-month event-free-survival. An event is defined as grade 3 or grade 4 toxicity, objective tumour recurrence, or death.

**Discussion:**

The aim of this clinical trial is to evaluate de-escalation of the CapRI-scheme. It is hypothesised that removal of cisplatin and radiotherapy will have no significant effect or only a minor impact on the clinical response but result in substantially lower toxicity.

**Trial Registration:**

Current Controlled Trials ISRCTN79802092

## Background

Only 10–20% of patients with pancreatic cancer can be resected with curative intention at the time of diagnosis. Unfortunately, loco-regional recurrence and/or metastatic disease develop in the majority of patients who undergo pancreatic resection. Most patients typically relapse within 9–15 months form initial presentation and have median life expectancies of only 12–15 months without adjuvant therapy. The 5-year survival of patients with resected pancreatic adenocarcinoma is approximately 14% for patients without adjuvant therapy [1]. Data from two randomized clinical phase III trials showed that adjuvant chemotherapy results in significant increase of overall survival (OS) and disease-free survival (DFS) [2,3]. Therefore, adjuvant therapy with either 5-FU or gemcitabine is recommended according to the German S3 guidelines for exocrine pancreas carcinoma [4].

Investigators form the Virginia Mason Clinic reported data from a phase II trial where in an adjuvant setting cisplatin, 5-FU, interferon-α2b (INF-α2b) and external beam radiation were administered. They reported from 43 patients overall 5-year survival data of 55% [5]. The randomized, open, controlled, prospective, multi-centre phase III CapRI-trial was initiated in August 2004 to confirm the excellent results of the CapRI-regimen [6]. The last patient was enrolled end of 2007. First clinical results are expected for 2009. ACOSOG and the group of Linehan et al. confirmed in phase II trials the results from the Virginia Mason Clinic to a certain extent [7,8]. These excellent results could be ascribed to the several synergistic effects between the combined substances. Radio-sensitising properties of 5-FU and cisplatin are well known. The incorporation of INF-α2b into a combined modality treatment program seems to offer a number of theoretical advantages. These include: 1) the radio-sensitization effects of INF-α2b and 5-FU [9,10]; 2) enhanced 5-FU based bio-availability; 3) a synergistic inhibition of pyrimidine metabolism with 5-FU and 4) an independent immunomodulatory effect of INF-α2b [11]. Furthermore, our own data derived from the translational program accompanying the CapRI trial showed especially immunomodulatory and anti-angiogeneic effects of INF-α2b [12-17].

However, especially the ACOSOG group reported severe gastrointestinal toxicities (93%) which led to enrolment stop. Virginia Mason Clinic reported 70% grade 3 or 4 toxicity and the group from the Washington-University stated 36% gastrointestinal toxicity. Although the final safety analysis is not yet performed we could give account on less GI-toxicity in the CapRI-trial. Nevertheless, the regimen is challenging and reduction of side effects is a useful goal.

## Methods/Design

### Trial organisation and coordination

CapRI-2 is designed and coordinated by the Department of Surgery, University of Heidelberg and the National Center for Tumor Diseases, Heidelberg (NCT). Heidelberg is responsible for overall trial management, regulatory affairs, statistical planning and analysis, trial registration and reporting as well as quality assurance. The responsibility for external monitoring and pharmacovigilance is carried forward to independent Contract Research Organisations (CRO). The trial will be conducted by a German network for pancreatic carcinoma (PC-Net) including the University hospitals of Munich (Technische Universität), Marburg, Ulm, Greifswald, Halle and Heidelberg. Further study centres will be recruited. The trial is sponsored by a private person, Dr. Wild from Germany. The financial sponsor is not involved in the database management and has no access to the randomisation code.

### Investigators

Patients will be recruited by the centres who will commit their participation. Due to the multimodal nature of the trial, all investigators will either be experienced oncologists, gastroenterologists, radio-oncologists or surgeons.

### Data Safety and Monitoring Board

An independent Data Safety and Monitoring Board (DSMB) consisting of three experts (one expert in oncology, one in pancreatic carcinoma and one in biometry/biostatistics) will evaluate the clinical research data on an ongoing basis to assure patient safety and study integrity for the study. The board will monitor the trial data, in particular the safety data, and makes recommendations based on the periodically reviewed data. Responsibilities are laid down in a DSMB Charta.

### Medication supply

All chemotherapeutic and immunotherapeutic agents will be prepared and provided by the corresponding pharmacy. Medication will be prepared for each patient specifically and delivered just prior to administration to the outpatient's department.

### On-site Monitoring

Monitoring on site will be performed according to good clinical practice (GCP) guidelines. Monitoring will be done by personal visits from clinical monitors according to the SOPs of the independent, external CRO. Monitors will review the entries into CRFs on the basis of source documents (30% source data verification). Data management will be performed by the same CRO. Pharmacovigilance is outsourced to a further CRO specialized on safety issues. Both data bases will be aligned at the end of the trial.

### Ethics approval and informed consent

The final protocol was approved by the ethics committee of the University of Heidelberg; Medical School (AFmu-071/2008) at May 16^th ^2008. This clinical trial complies with the Helsinki Declaration from 2004, the Medical Association's professional code of conduct, the principles of GCP guidelines and the Federal Data Protection Act. The trial will also be performed in keeping with local legal and regulatory requirements. The medical secrecy and the Federal Data Protection Art will be followed.

Written informed consent will be obtained from each patient in oral and written form before inclusion in the trial and the nature, scope and possible consequences of the trial have been explained by a physician in detail. The investigator will not undertake any measures specifically required only for the clinical trial until valid consent has been obtained.

### Patient selection

CapRI-2 focuses on hospitalised patients over 18 years of age with resected pancreatic adenocarcinoma during a 24 months period and started in October 2008. Men and women over 18 years of age with histological proven R0 or R1 resected [18] pancreatic adenocarcinoma will be screened for participation in the clinical trial. Patients will be contacted first-time by the (sub-) investigators either during their postoperative hospital stay or after discharge at the outpatient's department. A detailed overview of recruitment criteria for inclusion and exclusion is given in table 1.

**Table 1:** Eligibility Criteria

Inclusion criteria	Exclusion criteria
• R0/R1 resected pancreatic ductal adenocarcinoma	• Metastatic disease
• Adequate lab parameters (bone marrow-, liver and kidney function; Hb >8.0 g/dl, WBC >3,000 cells/mm^3^, platelets >75,000 cells/mm^3^; ALT/AST ≤ 2 ULN; Creatinine ≤1.5 mg/dL and calculated or measured creatinine clearance (CrCl) of ≥ 60 ml/min).	• Previous chemo- or radiotherapy for pancreatic carcinoma
• Therapy starts within eight weeks after surgery	• Previous radiotherapy in the corresponding region
• Ability of patient to understand character and individual consequences of clinical trial	• Patients with known severe depression
• Written informed consent must be available before enrolment in the trial	• Patients with severe heart diseases (NYHA stadium three and four) or severe lung disease (COPD Grade III, Asthma bronchiale Grade IV)
• For women with childbearing potential, adequate contraception.	• General condition worse than ECOG 2
• Age ≥ 18 years	• Pregnancy and lactation
	• History of hypersensitivity to the investigational product or to any drug with similar chemical structure or to any excipient present in the pharmaceutical form of the investigational product
	• Any contraindication met for any investigational product
	• Patients with mental diseases ICD-10-code F30, F31, F32.2 ff. or F33.2 ff.
	• Participation in other clinical trials and observation period of competing trials, respectively.
	• Serious uncontrolled acute infections at the time of therapy initiation or patients with known HIV infection, other immunodeficiencies or autoimmune diseases

### Study design

CapRI-2 is an open, controlled, prospective, randomized, multicentre phase II trial with three parallel study arms. Patients in study arm A receive a combination of 5-FU, cisplatin and external beam radiation including INF-α2b administration (CapRI). The first de-escalation step is in study arm B where cisplatin is omitted (CapRI-light). The second de-escalation will be tested in study arm C where beside cisplatin also radiotherapy is cancelled (CapRI-ultra-light). These treatments are offered to a heterogeneous group of people under clinical circumstances, covering a wide range, for both sexes and with heterogeneous characteristics/co-morbidities. No interim analysis is planed for this trial.

### Study objectives

Primary objective is the comparison of the treatment groups with respect to six-month event-free survival. An event is defined as tumour recurrence, grade 3 or grade 4 toxicity (according to CTCAE Version 3.0), or death (whichever occurs first). Secondary objectives are comparison of the treatment groups with respect to safety, OS, Recurrence-Free Survival (RFS), Quality of Life (QoL), screening for predictive markers, and accompanying translational research focussing mainly on immunological parameters.

### Randomisation

All patients enrolled will be identifiable throughout the study. The investigator will maintain a personal list of patient numbers and patient names to enable records to be found at a later date. Upon inclusion each patient receives a unique identification number. After the patient's eligibility for randomisation has been assessed he/she will be randomly assigned to one of the three treatment arms (1:1:1 randomization) and receive a unique randomisation number. By the randomisation number, it is possible to identify the order in which the patients entered the randomized study period at the centre in question. Randomisation will be stratified by centre; it will be done and documented centrally and send by fax. The randomisation list will be kept in safe and confidential custody at the involved CRO. The method of randomisation (whether fixed block size or randomly determined block size) and the block sizes themselves will not be disclosed to the study centres, the monitors or any other person involved in the study conduct. This information will be kept with the randomisation list.

### Treatment scheme

**Figure 1:**
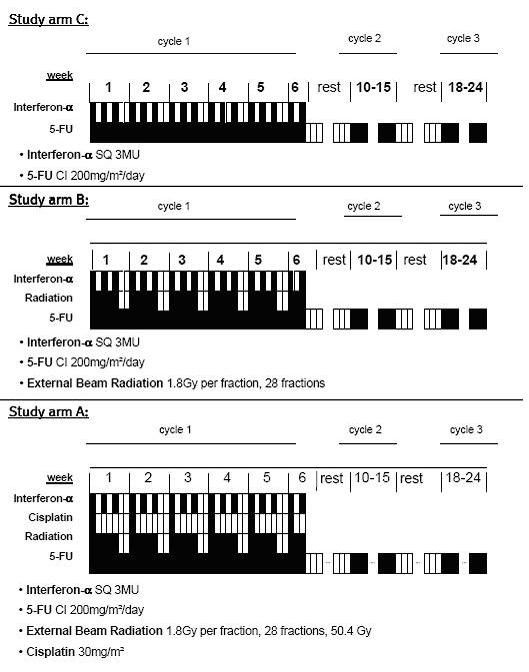
**Treatment scheme**.

After implantation of a Port-A-Cath catheter patients will be treated as follows (see figure 1): All arms: 200 mg/m^2^/day 5-FU by continuous intravenous infusion on days 1–38; 64–101, and 120–161; three million units IFN-α2b subcutaneous three times weekly days 1–38 plus one injection prior to treatment start given during week -1 (approx. day -6). Non-steroidal anti-inflammatory drugs and steroids should be avoided if possible during IFN-α2b treatment. Only arm A and B: Beside 5–FU and IFN-α2b patients will be treated with external beam radiation: The pancreatic bed will be covered with a minimum margin of 2 cm. The porta hepatis, origins of the celiac axis and superior mesenteric artery will be included. The fields must include the entire duodenal C-loop as seen on pre-operative CT scan. Total dose will be 50.4 Gy in 28 fractions over 5.5 weeks (1.8 Gy/day). Simulation should be done with the patient in the supine position with "arms up" position. A treatment planning CT is required to allow 3-D conformal treatment planning. Conventional 3-D treatment techniques as well as intensity modulated radiotherapy (IMRT) could be used for radiotherapy. At least a four field technique for 3-D planning or a multi-field technique for IMRT is necessary. Furthermore a multi-leaf collimator is required to allow customized blocking and IMRT. Equipment: greater than or equal to 6 MeV photons should be used. Only study arm A): Cisplatin 30 mg/m^2 ^IV over 60 minutes on days 1, 8, 15, 22, 29, 36 (6 doses). Two to three hours before and after Cisplatin dose the patients will receive hydration of at least 2 litres. Patients will be taught to drink at least 1 litre during the day.

### Evaluations, Toxicity-Based Dose Adjustment and follow-up

Pre-treatment evaluation for patients who are enrolled includes a single low dose (3 Mio U) injection of INF-α2b prior to therapy. Blood will be drawn for intensive immunological studies. All patients must have appropriate lab and radiographic studies conducted prior to study enrolment to meet eligibility criteria. Lab parameters will be obtained at least weekly and imaging will be performed every three months. The patients will be asked at each visit for any adverse event (AE) and concomitant medication. Quality of life (QoL) questionnaires (QLQ-C30 and the pancreas-specific Pan 26 questionnaire) will be handed out to the patients prior to therapy and after each cycle of chemotherapy. Samples for translational research (blood, plasma and urine) will be collected at the indicated time points (prior and 24 hours after first IFN-α2b administration, immediately prior to therapy start, first day of week 3 and at staging visits) and either analyzed immediately or kept frozen at -80°C until further analysis. Samples will be collected at each site and shipped on dry ice twice a year to Heidelberg.

Decisions regarding weekly chemoradiation treatment and chemotherapy dose-adjustment will be made using the guidelines below and based on haematological parameters monitored twice weekly during chemo-(radio)therapy. In case of grade 3/4 haematological toxicities chemotherapy should be held until resolution or until toxicity has resolved or dropped to a Grade 2 level. Radiation will be continued. IFN-α2b will be stopped in case of grade 4 haematological toxicities. The procedure for patients with leukocytes between 1.0 and 1.5/nl will be discussed individually in the study group. In case of grade 3/4 GI toxicities (including anorexia, dehydration, nausea/vomiting, diarrhoea, mucositis and bleeding) patients will be considered for periodic outpatient intravenous rehydration with anti-emetics according to conventional practice guidelines. Corticosteroids should be avoided. Any grade > 2 oto-, neuro-, or nephrotoxicity should lead to discontinuation of cisplatin until recovery to at least grade 1. All uncertain cases of toxicities will be discussed by the study group and a decision will be made according to the study group discretion. Patients will be investigated weekly for mental state. In case of psychological features, a psychiatrist will be consulted. Patients with mental diseases will be excluded.

Patients can withdraw from study participation at any time. Patients are taken off the study if unacceptable toxicity appears. Unacceptable toxicity is defined as serious side effects or irreversible Grade 4 toxicity independent whether they are expected or unexpected. Patients with mental diseases will be excluded as mentioned in exclusion criteria. Patients who withdraw from the study may be treated with 5-FU and folinic acid or with gemcitabine. The decision will be based on the individual reason of study withdrawal. Treatment with gemcitabine is recommended to patients with recurrence of disease. At the moment, no further therapy is recommended after completion of adjuvant therapy. Patients will undergo imaging and lab analysis (including tumor marker) quarterly and will be tracked by quarterly phone follow-up until death.

### Statistical considerations and sample size estimation

The primary endpoint in this clinical trial is the event-free survival. An event is defined as time from resection to objective tumour recurrence, grade 3 or grade 4 toxicity (according to CTCAE Version 3.0), or death (whichever occurs first). Secondary endpoints are as follows: a) OS, defined both as time from randomization and resection to death; b) RFS, defined as either time from randomization or as time from resection to relapse of death from any cause (whichever occurs first); c) QoL; d) Translational research focussing on immunological and angiogenic parameters, and e) safety issues.

For each parameter, several (longitudinal) measurements will be available. Apart from the parameter values themselves, further parameters will be generated, such as differences, or relative differences, of values after vs. before administration of INF-α2b or binary variables obtained by applying threshold values. Sample size calculations were based on the three-group comparison (differences in the three 6-month event-free rates r1, r2, r3 corresponding to CapRI (Arm A), CapRI light (Arm B), and CapRI ultra light (Arm C), respectively and carried out by means of computer simulations using the following main assumptions: equal group sizes, failure times arising from an exponential distribution, individual follow-up duration of ≥ 6 months, duration of patient enrolment 6, 12, or 18 months, For r1 = 2%, r2 = 10%, r3 = 15% the minimal sample size leading to an estimated power of ≥ 80% was 37 per group. Similarly, for r1 = 2%, r2 = 5%, r3 = 15% the necessary sample size estimated by the simulations was 38 per group. From the experience gained during the CapRI trial it can be expected that about 15% of the patients refuse further participation in the trial (including further follow-up examinations) once the result of the randomization has been disclosed to them. These patients do not contribute any information to the endpoints of this trial. In order to accommodate for a maximum drop-out rate of 15% the total sample size is therefore increased to 135. The main evaluation will be performed two years after the last patient's enrolment.

The assessment of safety will be based mainly on the frequency of AEs and on the number of laboratory values that fall outside of pre-determined ranges and/or show prominent worsening from baseline. AEs will be summarized by presenting the number and percentage of patients having any AEs or serious adverse events (SAE) and having each individual AE, and by determining and summarizing the maximum individual toxicity grade (over all forms of toxicity) for each treatment cycle. Furthermore, the most common AEs (those occurring in at least 10% of the treatment group) will be determined. Any other information collected (e.g. severity or relatedness to study drug) will be listed as appropriate. Laboratory data will be summarized by presenting shift tables using normal ranges (baseline to most extreme post-baseline value) and by presenting summary statistics of raw data and change from baseline values (means, medians, standard deviations, ranges). The analysis of safety and tolerability will be based on all patients entered into treatment who received at least two cycles of the systemic study therapy (= per protocol population of this study). All proportions will be given along with exact Pearson-Clopper 95% confidence bounds.

## Discussion

Only 10–20% of patients with pancreatic cancer can be resected with curative intention at the time of diagnosis [19]. The 5-year survival of patients with resected pancreatic adenocarcinoma is approximately 14% for patients without adjuvant therapy [1]. Data from two randomized clinical phase III trials showed that adjuvant chemotherapy results in significant increase of overall survival and disease-free survival [2,3]. The Virginia Mason study group in Seattle, USA, published very promising data in a phase II trial involving immunotherapy and chemo-radiation in the adjuvant setting [5]. The reliability of the data has been intensively discussed and therefore a source-data-verification was performed by the National Cancer Institute (NCI). Finally, the reference adjuvant treatment regimen from a RCT [2] and the very promising data from a phase-II trial [5] were the basis for the randomized, controlled multicentre CapRI-trial [6]. First clinical results are expected for 2009.

So far, the observed toxicity in the CapRI trial is less than reported from the Virginia Mason Clinic and other groups in USA. This might be due to differences in radiation treatment and supportive care during chemotherapy. However, the CapRI-scheme is nonetheless a quite toxic regimen. In our experience haematological side effects dominate, followed by gastrointestinal toxicity. Most of the toxicities are thought to be related to cisplatin. Furthermore, *in vivo *data indicate that the combination of 5-FU and INF-α2b is responsible for the anti-tumour effect [12,13,16,17]. Moreover, there are data supporting the idea that radiotherapy is of low effectiveness [2,20,21]. Therefore, it is hypothesized that removal of cisplatin and radiotherapy will have no significant effect or only a minor impact on the clinical response but result in lower toxicity. The current clinical trial, CapRI-2, will investigate this hypothesis on the basis of a de-escalation of the CapRI-regimen. CapRI-2 is a phase II trial focussing on event-free survival. Obviously, it has to be expected that toxicity decreases when less chemotherapy is administered. However, as recurrence and death are defined as events too, some insights into clinical relevance of the de-escalating schemes could be made. As a phase II trial the comparison of OS can not be the primary objective of this trial and moreover, a trial design showing equality or non-inferiority regarding survival and superiority regarding toxicity would need thousands of patients. The results of CapRI-2 will bring hints whether and which de-escalation might be worth to be investigated in further trials. The results of the CapRI-2-trial will advance clinical and scientific knowledge on the adjuvant treatment of pancreatic adenocarcinoma as it may help to improve clinical results on the one hand and reduce therapeutic-related toxicities on the other.

## Abbreviations

5-FU: 5-Fluorouracil; AE: Adverse Events; CTCAE Version 3.0: Common Toxicity Criteria 3.0; CRF: Case Report Form; CRO: Contract Research Organisation; DFS: Disease-free survival; DSMB: Data and Safety Monitoring Board; GCP: Good Clinical Practice; GI: Gastrointestinal; NCI: National Cancer Institute; OS: Overall survival; QoL: Quality of Life; RCT: Randomized Controlled Trial; RFS: Recurrence-free survival; SAE: Serious Adverse Events

## Competing interests

The authors declare that they have no competing interests.

## Authors' contributions

AM, MWB, MM, DJ, HF, JM and JS participated in the design of the study, UA was responsible for the statistical planning of the trial, JO and SH wrote the study protocol. All authors read and approved the final manuscript.

## Pre-publication history

The pre-publication history for this paper can be accessed here:

http://www.biomedcentral.com/1471-2407/9/160/prepub
